# Isolation and Identification of Phyllospheric Bacteria Possessing Antimicrobial Activity from *Astragalus obtusifolius*, *Prosopis juliflora*, *Xanthium strumarium* and *Hippocrepis unisiliqousa*

**Published:** 2017

**Authors:** Zohreh Mazinani, Marzieh Zamani, Soroush Sardari

**Affiliations:** Drug Design and Bioinformatics Unit, Department of Medical Biotechnology, Biotechnology Research Center, Pasteur Institute of Iran, Tehran, Iran

**Keywords:** Antimicrobial agents, 16s rRNA, *Bacillus amyloliquefaciens*

## Abstract

**Background:**

The widespread utilization of antimicrobial compounds has caused emergence of resistant microorganisms in the world. Hence, the research to probe the products with antimicrobial features has led to finding natural habitats and discovering new pharmaceutical products.

**Methods:**

In this study, an attempt was made to explore the niche of novel habitat to isolate pyllospheric bacteria from the above ground parts (stems and leaves) of *Astragalus obtusifolius*, *Prosopis juliflora*, *Xanthium strumarium*, and *Hippocrepis unisiliqousa* to evaluate their antimicrobial features. The inhibitory effects of these strains on the growth of two fungi (*Aspergillus niger*, *Aspergillus fumigatus*), two yeasts (*Saccharomyces cerevisiae*, *Candida albicans*) and six bacteria (*Escherichia coli*, *Staphylococcus aureus*, *Pseudomonas aeruginosa*, *Bacillus subtilis*, *Salmonella typhi*, *Streptococcus pyogenes*) were tested.

**Results:**

In total, 113 bacterial strains were isolated. Twenty five bacterial strains (B-1 to B-25) indicated promising antimicrobial (antibacterial and antifungal) activities against aforementioned pathogens. The identification of the bacterial strains was ascertained by morphological, physiological, biochemical tests and two strains with the strongest antimicrobial activities were further characterized based on 16s rRNA sequencing. These two strains were identified as *Bacillus amyloliquefaciens*.

**Conclusion:**

Our results provide evidence that phyllospheric microorganisms are capable of producing some compounds with antimicrobial properties.

## Introduction

The aerial portions of plants such as buds, fruits and other above-ground parts as leaves and stems that make a habitat for microorganisms are called phyllosphere 1,2. It is an exclusive and dynamic environment that is believed to play a role in irregular and sometimes large changing of temperature, UV radiation and leaf humidity 3.

Microbes can be found on different segments of plants as epiphytes on the surface and as endophytes inside the plant tissues. The most important microbial inhabitants in phyllosphere are bacteria; however, the other microorganism populations such as archaea, filamentous fungi, and yeasts are considered to be significant and are present as well 1,4,5. These bacteria include both pathogenic and non-pathogenic population that contribute to health condition of the host plant and may even play global role in carbon and nitrogen cycles 6–9. It should be noticed that among nearly 300,000 available plant species living on earth that each is a host to at least one of these mentioned phyllospheric bacteria, only a little portion of them has been fully studied 10. Different bacteria have been isolated from different sources of plants such as trees (pins, yew), vegetables (carrot, tomatoes, soybean), fruits (citrus, pineapple) fodders (alfalfa, clover) and other crops as wheat and maize grains and coffee beans 11.

These bacteria have been isolated from woody plants such as gymnosperms and angiosperms and from flower, leaf, stem and fruit of different plant species as well 12,13. The contribution of these phyllospheric bacteria to various activities has been reported such as the contribution in defense mechanisms of plants 11, biological nitrogen fixation 14, increasing plant mineral absorption 15, decreasing disease severity 16,17 and inducing plant growth 18.

Moreover, it has been reported that endophyte bacteria can produce a number of metabolites and secondary metabolites such as antibiotics, antitumor compounds and plant growth inducing factors together with other factors like biological control agents, anti-inflammatory and biological control agents 19.

*Astragalus obtusifolius*, *Prosopis juliflora* and *Hippocrepis unisiliqousa*, families of *Fabaceae* are legumes that grow naturally in arid and semi-arid parts of the world. They have been observed in many tropical regions including Southeast Asia, South Asia, Northeastern Brazil, Australia and Africa. They have been reported to be resistant to salinity, heat and drought; and produce annually 10– 50 *kg* of pods used as a dietary resource for livestock and food resources for human. The flowers are small and individually invisible, both male and female, occurring in dense heads or clusters 20,21.

*Xanthium strumarium* is one of the members of *Asteraceae* family, an herb of several crops in the subtropical and temperate areas of the world, which is capable of invading watercourses, along beaches, railway and deserted places, roadsides, cultivated fields and edges, and dunes. It is an herbaceous annual plant, up to 2.5 *m* tall, and its leaves are broad, 15 *cm* in diameter and practically textured with some bristles 22,23.

Antimicrobial compounds are usually produced by *Bacillus* strains. *Bacillus* is a spore-forming bacterium and is capable to live in most habitants 24. The aim of this research was to investigate whether the internal tissues and external surfaces of four plant species *Astragalus obtusifolius*, *Prosopis juliflora*, *Xanthium strumarium* and *Hippocrepis unisiliqousa* are colonized by pyllospheric bacteria, to isolate and characterize phyllospheres and to test their antimicrobial features. This is the first report of isolation of phyllospheric bacteria from the cited plants.

## Materials and Methods

### Plant materials

Pyllospheric (endophytic and epiphytic) bacteria were isolated from *Astragalus obtusifolius, Hippocrepis unisiliqousa, Prosopis juliflora* belonging to the family of Fabaceae and *Xanthium strumarium* belonging to the family of Asteraceae. The above ground parts of plants were collected between 2007–08 from four locations in Khuzestan, Iran and transported to the laboratory. A total of 2 *kg* plant tissues were collected from each location.

### Isolation of bacterial strains

The collected plants tissues (stems, leaves) were washed under running tap water to remove soil particles. Samples were then dried for 7 days at room temperature and were powdered by electric mills. 0.02 grams of plant powders were added to 15 *ml* of sterile distilled water for homogenization. 2–3 *ml* of these plant solutions were aseptically transferred to plates containing tryptic soy agar (TSA, Sigma, Germany) medium 25. Benomyl (50 *mg/ml* of each) was added to the medium to suppress fungal growth 16. Plates were incubated for 4–7 days at 37°*C*. Isolated colonies were purified on TSA plates and pure strains were maintained on 20% (*v/v*) glycerol at −20°*C* for further studies 26.

### Antimicrobial activity assay

***Test organisms:*** Antimicrobial activity of the purified isolates was carried out against many bacterial pathogens and fungal pathogens, including Gram positive organisms *Staphylococcus aureus* (ATCC 25923), *Streptococcus pyogenes* (ATCC 8668), and *Bacillus subtilis* (PTCC 1365), and the Gram negative bacteria *Escherichia coli* (ATCC 25922), *Pseudomonas aeruginosa (P. aeruginosa)* (ATCC 27853) and *Salmonella typhi* (PTCC 1609); and the test fungi used were *Candida albicans* (ATCC 10231), *Saccharomyces cerevisiae* (BY 4743), *Aspergillus niger* (N 402) and *Aspergillus fumigatus* (AF 293) on Nutrient Agar medium (NA, HiMedia, Mumbai) and Sabouraud Dextrose Agar medium (SDA, Merk, Germany), respectively. All the reference strains were obtained from the Department of Microbiology, Pasteur Institute of Iran, Tehran. In the present study, a microbial lawn was made to culture the reference isolates. Afterwards, the bacterial isolates were placed onto the lawn made in NA and SDA plates. The plates were incubated at 37°*C* for 24 and 48 *hr* in the case of bacterial and fungal pathogens, respectively 27. They were observed for a zone of growth inhibition and the isolates which showed positive antimicrobial activities were selected for phenotypic and genotypic studies.

### Phenotypic characterization

The bacterial strains were identified to the genus-level based on the colony morphology (appearance, size, margin, form, elevation), microscopic examination (gram’s and endospore staining), physiological tests (growth at different NaCl concentrations, temperature and pH) and biochemical tests (catalase, oxidase, nitrate reduction, starch hydrolysis, casein hydrolysis, Voges Proskauer, citrate utilization, gelatin liquefaction, methyl red) by adopting standard procedures 16,28,29. The strains were coded and started with B-1, and the last number was B-25.

### Genotypic characterization

The genomic DNA of two bioactive isolates (having an inhibition zone against the majority of the test organisms) was extracted as described by Lee *et al* 30. The extracted DNA was diluted in sterile water and stored at −20°*C*. 16S rDNA amplification was performed using the universal primers 8F (5′-AGAGTTTGATCCTGGCTCAG-3′) and 1492R (5′-GGTTACCTTGTTACGACTT-3′) as described by Singh *et al* 31. The PCR reaction was performed for 30 cycles in a Thermal Cycler (Eppendorf, Germany), and the thermal program used for the PCR was under the following conditions: initial denaturation at 95°*C* for 2 *min*, final denaturation at 94°*C* for 30 *s*, primer annealing at 50°*C* for 30 *s*, extension at 72°*C* for 90 *s* and an addition of 10 *min* at 72°*C* as final extension. The PCR amplification was performed with a total volume of 20 *μl* containing 1 *μl* of DNA, 1 *μl* of 10 *pmol* 8F primer, 1 *μl* of 10 *pmol* 1492R primer (purchased from Generay Biotech, Shanghai, China), 10 *μl* of Master Mix (Vivantis) and 7 *μl* of sterile MilliQ water. PCR product was subjected to electrophoresis on 1.2 % (*w/v*) agarose gels in 1×TAE Buffer for 30 *min* at 90 *V*. The amplified product was visualized with UV-transilluminator. The nucleotide sequences of the 16S rRNA gene of isolates were determined using the dideoxy chain termination method utilizing big dye terminator ready reaction mix. The nucleotide sequences of the 16S rRNA gene of two isolates were registered in GenBank with accession number KM189814 (http://www.ncbi.nlm.nih.gov/nuccore/KM189814) and KM189815 (http://www.ncbi.nlm.nih.gov/nuccore/689584175).

**Figure 1.**
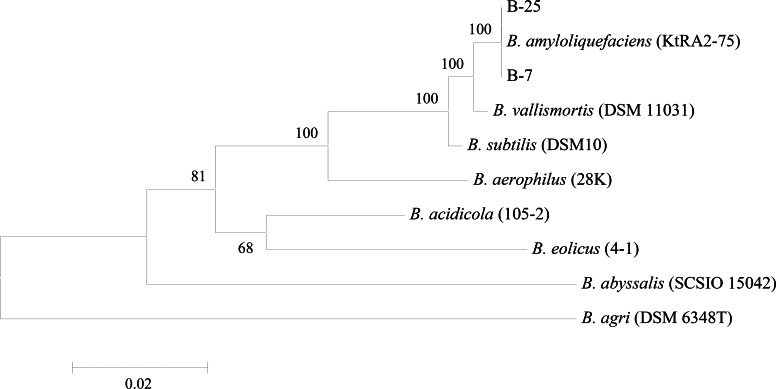
Phylogenetic tree based on 16S rRNA nucleotide sequences. The scale bar corresponds to 0.02-estimated nucleotide substitution per sequence position. Bootstrap values from 1000 replicates.

Multiple sequence alignments were carried out using MEGA 5 software (http://www.megasoftware.net/). For phylogenetic analyses, obtained sequences were compared to those available in the GenBank ( www.ncbi.nlm.nih.gov ) databases and a phylogenetic tree was constructed by the Neighbor Joining (NJ) algorithm 32 (Figure 1).

## Results

### Isolation of bacterial strains

Endophytic and epiphytic strains were observed emerging from the above ground parts of plants sampled 4–7 days after incubation. A total of 104 bacterial strains were isolated from the four plants species, among which 18 were from *Astragalus obtusifolius*, 32 from *Prosopis juliflora*, 31 from *Xanthium strumarium* and 23 from *Hippocrepis unisiliqousa*.

### Antimicrobial assay

**Figure 2.**
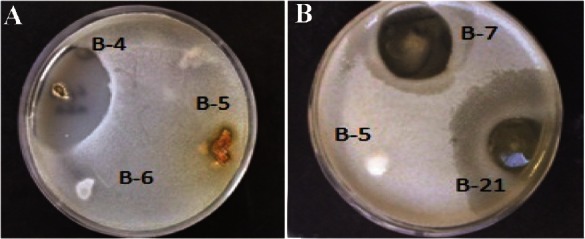
Zones of inhibition produced by a) strain B-4 against *B. subtilis* and b) strains B7 and B-21 against *C. albicans*.

Based on the results of antimicrobial activity (Table 1), 25 strains (26%) showed a zone of growth inhibition against one or more of the test organisms. Of 25 bioactive strains, 7 strains (B-1, B-3, B-4, B-7, B-12, B-21, and B-22) showed good antimicrobial activities against one or more of test pathogens. Among these strains, 3 (B-1, B-4 and B-22) had antibacterial activity and 4 (B-3, B-7, B-12 and B-21) showed broad antimicrobial activities from which 2 strains were selected for species identification. Interestingly, the isolate B-4 had potent antibacterial activity against *B. subtilis* (Figure 2). It is in accordance with the result of Hung *et al* 24 who indicated that out of 109 bacterial endophytes, 84% were capable of producing antimicrobial metabolites. In all microbial tests, standard comparison was done. The applied ATCC isolates were standard. Also, due to high reproducibility of the antimicrobial method, duplicate repeats were performed.

**Table 1.** Influence of bacterial strains isolated on the growth of the test organisms

**Test strains**	**Inhibition zone (in mm diameter) caused by the test organisms**									

	**Gram positive bacteria**			**Gram negative bacteria**		**Yeast**		**Fungi**	

	***S. aureus***	***S. pyogenes***	***B. subtilis***	***E. coli***	***S. typhi***	***C. albicans***	***S. cervisiae***	***A. niger***	***A. fumigatus***
**B-1**	−	−	−	+	−	−	−	−	−
**B-2**	+	−	−	−	−	−	−	−	+
**B-3**	+	+	+	+	−	+	+	−	−
**B-4**	+	−	+	−	−	−	−	−	−
**B-5, B-6, B-8**	+	−	−	−	−	−	−	−	−
**B-7, B-25**	+	+	+	+	−	+	−	+	−
**B-9, B-11**	−	+	−	−	−	−	−	−	−
**B-10, B-13**	−	+	−	−	+	−	−	−	−
**B-12**	−	+	−	+	+	+	+	−	−
**B-14**	−	−	−	−	+	−	−	+	−
**B-15, B-16**	−	−	−	+	−	−	−	+	−
**B-17**	−	−	−	+	−	−	−	+	+
**B-18**	+	−	−	−	−	−	−	+	+
**B-19**	−	−	−	+	−	−	−	−	+
**B-20, B-22, B-23**	−	−	+	−	−	−	−	−	−
**B-21**	−	−	+	−	−	−	−	−	+
**B-24**	−	−	−	−	+	−	−	−	−

### Phenotypic and genotypic characterization

Results of microscopic examination indicated that the selected isolates (B-7 and B-25) were Gram positive, motile, rod-shaped and endospore forming. The colonies were large, fast growing, wrinkled, and dry, white, irregular and flat on TSA medium incubated at 28°*C*. They grew well at 25–50°*C*, pH=6–9 and could tolerate up to 10% NaCl. Both of these isolates were positive for catalase, oxidase, nitrate reductase, citrate utilization, and starch- and casein-hydrolysis and negative for gelatin liquefaction, Voges-Proskauer, methyl red, and hydrolysis of urea tests. Phenotypic features of other isolates are shown in table 2 as described by Smibert *et al* 33. These features revealed that the selected isolates belonged to the genus *Bacillus*. The identity was further confirmed by 16S rRNA gene sequencing and obtained results indicated 100% similarity to *Bacillus amyloliquefaciens* (Figure 2).

**Table 2.** Biochemical and physiological characteristics of *Bacillus* strains

**Characteristics**	**B-1, B-2, B-17, B-18, B-20, B-21**	**B-3, B-7, B-9, B-13, B-22, B-23, B-25**	**B-14, B-24**	**B-4, B-15**	**B-6**	**B-5, B-8, B-10**	**B-12, B-16, B-19**	**B-11**
**Gram’s staininig**	+	+	+	+	+	+	+	+
**Endospore formation**	+	+	+	+	+	+	+	+
**Growth at**	−	−	−	−	−	−	−	−
**10 °*C***	+	+	+	+	+	+	+	+
**25 °*C***	+	+	+	+	+	+	+	+
**30 °*C***	+	+	+	+	+	+	+	+
**37 °*C***	+	+	+	+	+	+	+	+
**50 °*C***	+	+	+	+	+	+	+	+
**65 °*C***	−	−	−	−	−	−	−	−
**Growth at pH range**								
**6.0**	+	+	+	+	+	+	+	+
**7.0**	+	+	+	+	+	+	+	+
**8.0**	+	+	+	+	+	+	+	+
**9.0**	+	+	+	+	+	+	+	+
**Growth in the presence of**								
**3% NaCl**	+	+	+	+	+	+	+	+
**5% NaCl**	+	+	+	+	+	+	+	+
**7% NaCl**	+	+	+	+	+	+	+	+
**10% NaCl**	+	+	+	+	+	+	+	+
**Hydrolysis of**								
**Casein**	+	+	+	+	+	+	+	+
**Starch**	−	+	−	+	−	+	+	+
**Urea**	−	−	−	+	−	−	−	+
**Biochemical tests**								
**Catalase**	+	+	+	+	+	+	+	+
**Oxidase**	+	+	+	+	+	+	+	+
**Nitrate reduction**	−	+	+	+	−	+	+	+
**Gelatin liquefaction**	−	−	−	−	−	+	−	−
**Citrate utilization**	+	+	+	+	−	−	−	−
**Methyl Red (MR)**	−	−	−	−	−	−	−	−
**Voges Proskauer (VP)**	−	−	−	−	−	−	−	−

## Discussion

Several authors have reported the presence of various bacteria species in plant samples, such as *Bacillus pumilus* (as dominant endophytes) in citrus plants 34, *Stenotrophomonas* sp. in sweet potato plant 35 and in the coffee seed 36, *Pseudomonas putida* in carrot 37, and *Seratia marcescens* isolated from rice 38. Our results indicated that bacillus can be obtained as epiphytes both inside leaves and stems tissues. It is in agreement with the obtained results by Ryan 39 and Dudjea *et al* 40 who reported the existence of *Bacillus* species and other endophytic bacteria on legume plants. The presence of bacillus generally inside the leaves and stems tissues plays a significant role in plant growth and health 41. Dini Andreote *et al* 42 reported bacillus’ potential in controlling plant diseases and its ability in production of antimicrobial agents. In the present work, the presence of *Bacillus* in leaves and stems of two plant families from a tropical area was confirmed, as it was reported by Deng *et al* 43.

In this study, none of the strains was shown to be bioactive against *P. aeruginosa.* Antimicrobial studies of *bacillus* from plant tissues are rare. Goryluk *et al* 44 isolated 34 *bacillus* isolates from the above ground parts of *Chelidonium majus* in Warsaw, Poland, among which 19 exhibited antimicrobial properties. Only one isolate showed antifungal activity against all the tested organisms. Suciatmih *et al* 45 obtained 153 endophytic bacterial isolates from 67 plant species in West Java, Indonesia. 31 isolates exhibited excellent antimicrobial activities against test fungal isolates.

Genotypic analysis is in agreement with the obtained results by Ma *et al* 46 who isolated 104 endophytic bacteria from different tissues of Panax notoginseng and observed that the most major species in all parts of plants were *Bacillus amyloliquefaciens* subsp. Moreira *et al* 28 initially isolated *Bacillus* species from several different plants and identified them on the basis of the 16S rDNA sequence as *Bacillus amyloliquefaciens*. Chauhan *et al* 47 showed the predominance of *Bacillus* in different sugarcane varieties. These *Bacillus* strains were reported to have important antifungal activity. Athukorala *et al* 48 reported that *Bacillus* strains, from different microhabitats in Canada, had important antifungal activities. A novel *Bacillus* spp. AB1, with strong antifungal activity, was obtained from coffee phyllosphere of the Nile River in India by Nair *et al* 49. Muzzamal *et al* 32 obtained *Bacillus* isolates from different plant tissues (stem, root, fresh and wilted leaves) in Punjab, Pakistan which were active against both Gram positive and Gram negative bacterial isolates. Wang *et al* 50,51 reported the significance of water habitats as the source of bioactive *Bacillus*. They obtained *Bacillus* species from paddy fields in China. The culture suspension of this isolate was found to exhibit potent antifungal activity against plant pathogens.

## Conclusion

As antibiotic resistance increases among human pathogenic organisms, there is an urgent need for development of new antibiotics. In this regard, exploring new sources of antibiotics is extremely important. Among the new sources, phyllospheric microorganisms that are capable of producing some compounds with antimicrobial properties are the typical ones.
